# Early Bone Healing on Hydroxyapatite-Coated and Chemically-Modified Hydrophilic Implant Surfaces in an Ovine Model

**DOI:** 10.3390/ijms22179361

**Published:** 2021-08-28

**Authors:** Elnaz Ajami, Cong Fu, Hai Bo Wen, Jeffrey Bassett, Sun Jin Park, Marie Pollard

**Affiliations:** Department of Research and Development, Zimmer Biomet Dental, Palm Beach Gardens, FL 33410, USA; Elnaz.Ajami@zimmerbiomet.com (E.A.); henryfu1985@gmail.com (C.F.); HaiBo.Wen@zimmerbiomet.com (H.B.W.); Jeffrey.Bassett@zimmerbiomet.com (J.B.); Jenny.Park@zimmerbiomet.com (S.J.P.)

**Keywords:** peri-implant bone, osseointegration, hydroxyapatite, histomorphometry

## Abstract

Implant topography affects early peri-implant bone healing by changing the osteoconduction rate in the surrounding biological environment. Implant surfaces have been designed to promote faster and stronger bone formation for rapid and stable prosthesis loading. Early peri-implant bone healing has been observed with a sandblasted, acid-etched implant that was chemically modified to be hydrophilic (cmSLA). The present study investigates whether early peri-implant bone healing extends to a rough surface implant with a high crystalline hydroxyapatite surface (TSV MP-1 HA). Three implants were randomly placed in porous trabecular bone within both medial femoral condyles of 10 sheep. Early peri-implant bone stability was measured at 3- and 6-weeks healing time following implant insertion. Results indicated a similar implant stability quotient between the implants at insertion and over time. The significant increase over time of reverse torque values with respect to insertion torque (*p* < 0.001) did not differ between the implants. However, the bone-to-implant contact of TSV MP-1 HA was significantly higher than that of cmSLA implants at 6 weeks (*p* < 0.01). These data validate previous findings of a hydrophilic implant surface and extend the observation of early osseointegration to a rough surface implant in porous trabecular bone.

## 1. Introduction

Dental implantation triggers a cascade of events, starting with the formation of a hematoma or clot formation. This is followed by an inflammatory response and angiogenesis that provide a pathway for the migration of osteogenic cells towards the implant surface (i.e., osteoconduction). During the process, the implant topography can influence the retention of a variety of proteins that aid in osteoconduction [1,2]. For example, non-collagenous proteins are secreted by differentiating osteogenic cells that reach the implant surface. In turn, non-collagenous proteins initiate calcium phosphate nucleation followed by crystal growth. The process continues with the assembly and compartmental calcification of collagen fibers, leading to the anchoring of newly formed bone to the implant. If the implant surface features a multi-dimensional complex topography, then the bone bonding and interdigitation with the implant surface is enhanced [2]. Improving the rate and extent of osseointegration in dental implantology by means of altering surface properties of dental implants has been one of the most widely investigated areas over the past few decades. With the advancements in the field, various technologies have been developed to improve implant surface topography (roughness), chemistry, and surface energy in order to enhance and accelerate the peri-implant bone healing process [3]. These technologies, which can generally be grouped into physical (e.g., plasma spray coating; grit blasting) and chemical (e.g., acid- or alkaline-etching; anodizing) surface modifications [4], have been used by many dental implant companies worldwide and resulted in significant improvements in peri-implant bone healing compared to the unmodified (turned) implant surfaces [5]. Titanium and its alloys are particularly suitable for anodization, which allows for ease of chemical surface modification via metal etching and oxide growth. The anodization process can be controlled to produce both a titanium oxide layer of porous nanotubes and additives to promote osseointegration [6].

Among the physical surface treatment techniques, plasma-sprayed hydroxyapatite (HA) coating is one of the earliest techniques applied on dental implants and has been used clinically since 1984 [7]. However, there are contradictory reports regarding the benefits and the coating integrity of the plasma-sprayed HA-coated implants [8]. The benefits of such coating in enhancing bone-to-implant contact (BIC) as compared to machined surfaces are widely accepted through numerous studies [9,10,11,12]. However, there are controversies regarding the long-term stability and delamination of the coating with low crystallinity [7,13,14], as high amounts of amorphous phase HA could be subject to a rapid dissolution under certain adverse conditions [15]. To address this issue, a post-plasma spray that is pressurized and hydrothermal is applied to convert the HA coating to a highly crystalline surface that has a dense microstructure with low impurity and high bonding strength [16].

In the 2000s, a surface technology was developed by a chemical technique that involved sequential sandblasting and acid-etching (SLA) to create a complex implant microtopography. The complex implant surface was further treated with nitrogen gas followed by saline that resulted in a hydrophilic chemically-modified (cmSLA) surface [17]. The cmSLA implant has been reported to facilitate the rate of osseointegration [18] by enhancing the recruitment and migration of pre-osteogenic cells to the implant surface [19]. In an in vivo canine mandibular model, the cmSLA surface achieved significantly faster osseointegration (2 weeks) as compared to its non-hydrophilic SLA version (4 weeks), implying that patients undergoing early loading procedures might receive a beneficial reduction in the healing time with the cmSLA implant [20]. In another in vivo study [21], an increased early bone apposition within 2 weeks of healing was observed with the same type of hydrophilic implants placed in the mandible or maxilla of four canines. Moreover, faster osseointegration characteristics of the hydrophilic implant type were observed in an ovine model, where the cmSLA and SLA implant types were placed in the tibiae of three sheep. The cmSLA implant type achieved a higher bone contact and stability at a 3-week healing period [22]. Despite the advantage of the cmSLA, the authors noted that both implant types resulted in excellent outcomes that appeared to be due to the dense cortical structure of the tibia [22]. This experimental ovine model was previously used for testing various dental implant surfaces. Compared to other animal models, the ovine bone turnover and remodeling rates were closer to that of human bone in areas outside the oral cavity, which are less prone to infection [23,24,25].

We aim to assess whether the early peri-implant bone healing observed for the cmSLA implant extends to an implant with a plasma-sprayed and highly crystalline hydroxyapatite surface (TSV MP-1 HA). The commercially available cmSLA and TSV MP-1 HA implants were tested concomitantly in the ovine femoral condyle model to validate the experimental set-up under different conditions from the previous ovine tibia model. The translational value of the ovine model was supported by various similarities with humans. For instance, sheep and humans show similarities in body weight, mechanical and physical bone properties [26], and the pattern of distal femoral bone ingrowth into porous implants [27]. The International Standard Organization (ISO 10993-6) recommends the use of larger animals for implantation of 4.5 mm diameter and 12 mm length implants into the femur and tibia. As such, sheep are also suitable for investigating standard size dental implants used in the present study and in the clinic [25,28]. Although dogs show more similarity in bone composition to humans than sheep, there is a large variation between dogs in trabecular bone turnover [29]. Therefore, the ovine model was considered to be a very reliable choice in terms of consistency in measurements and translation to the clinic. The choice of bone site was based on the prevalence of trabecular porous bone encountered in the femoral condyles of sheep [27]. The authors of a previous study noted that the excellent outcomes for both SLA and cmSLA may have been attributable to the dense cortical structure of the tibia bone [22]. Therefore, the porous nature of trabecular bone was considered to be a challenging site for investigating dental implant stability. Baseline measurements of the implant stability quotient (ISQ) using resonance frequency analysis (RFA) and insertion torque values (ITV) were established to ensure adequate initial stability as a prerequisite for early bone healing and successful osseointegration. The reverse torque values (RTV) were determined with respect to the baseline ITV, which depends on the site preparation protocol that is suitable for each implant macrogeometry, as established by the manufacturer. The aforementioned mechanical measures (ISQ, RTV) as well as histological evaluations of BIC% and bone area fraction (BAF%) were subsequently obtained after a healing period of 3 and 6 weeks. The expectations of the current study were a lack of statistical difference in early osseointegration between cmSLA and TSV MP-1 HA dental implants, given that both have a much greater surface roughness compared to metal implants with smooth surfaces [5]. Early peri-implant bone healing manifested as increases in RTV over time for both implant systems and a larger BIC% at 6 weeks for TSV MP-1 HA implants. The results of this study will serve to advance knowledge in microgeometric designs that promote efficient bone regeneration and integration for dental treatment. The promotion of early implant osseointegration by the bone mineral-like hydroxyapatite coating is particularly beneficial for implantation in areas with lower bone density [30].

## 2. Results

The post-operative period was uneventful for all the animals, except for minimal swelling over all incision sites, which was sometimes accompanied by fluid accumulation within the overlying tissues. Although minimal fibrous tissue formation around the implants was initially observed, the surgical sites appeared to heal well by the completion of both healing periods. There were no implant failures, and none of the implants harvested at 3 and 6 weeks exhibited adverse tissue responses.

### 2.1. Implant Surfaces

**Figure 1:**
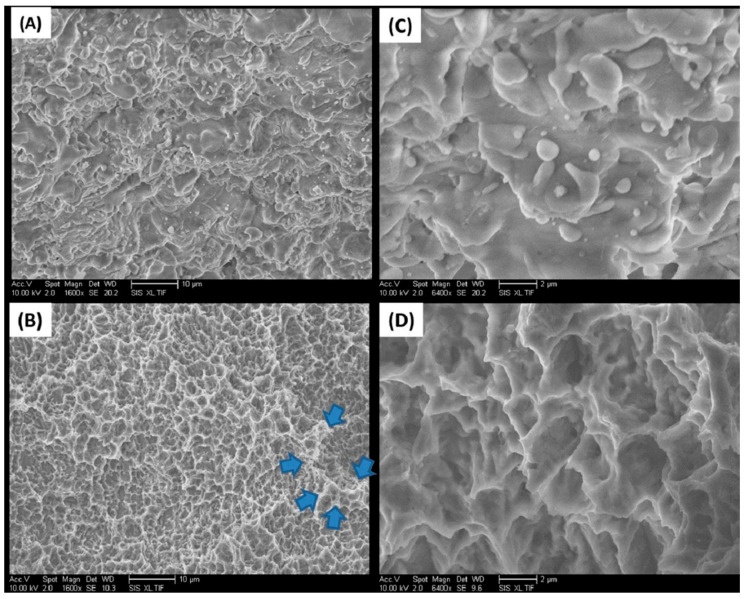
SEM images of TSV MP-1 HA (**A**,**C**) and cmSLA (**B**,**D**) implant surfaces at a magnification of 1600× at a 10 µm scale (**A**,**B**) and 6400× at a 2 µm scale (**C**,**D**). Arrows in (**B**) indicate approximately 1–4 µm pits superimposed on larger craters.

Scanning electron micrographs were consistent with distinct differences in the surface morphology of the implant systems (Figure 1). The TSV MP-1 HA implant surface appeared to be fully covered by a heterogeneous HA coating morphology. The appearance is due to the application of HA as molten particles that fuse to the surface of the TSV MP-1 HA implant (Figure 1A). In contrast, the cmSLA implant appears as a highly complex topography of pits superimposed on large craters that result from acid etching and grit blasting, respectively (Figure 1C). The representative EDX analysis (Table 1) revealed specific peaks on the TSV MP-1 HA implant attributed to Ca and P, which were absent on the cmSLA implant. The cmSLA implant only had peaks attributed to Na, Cl, and Ti due to the immersion in saline. The average surface roughness (R_a_) and standard deviation of TSV MP-1 HA and cmSLA implant surfaces were measured as 4.63 ± 0.83 and 1.57 ± 0.24 µm, respectively in an area of 50 × 1939 µm^2^.

### 2.2. Resonance Frequency Analysis

At the time of implant placement, the ISQ between the two implant systems and healing groups was similar and indicative of primary stability (Table 2). In group A (3 weeks), the median and interquartile range (IQR) of ISQ at insertion was 69.2 (2.8) and 70.0 (6.9) for TSV MP-1 HA and cmSLA, respectively. In group B (6 weeks), the median (IQR) of ISQ at insertion was 66.5 (5.6) and 68.4 (8.7) for TSV MP-1 HA and cmSLA implants, respectively. At the 3- and 6-week healing time points, the change in ISQ with respect to the baseline insertion values significantly increased for cmSLA implants at both time points (*p* = 0.02 and *p* = 0.05, respectively; Table 2). A significant increase in ISQ for TSV MP-1 HA was only observed at the 6-weak healing time (*p* = 0.03; Table 2). However, these changes in ISQ from baseline did not differ significantly between the implant groups at 3 and 6 weeks (*p* = 0.08 and *p* = 0.75, respectively).

### 2.3. Torque Testing

At the time of implant placement, the ITV of TSV MP-1 HA implants at a median (IQR) of 102.7 (61.2) Ncm was significantly higher (*p* = 0.01) than that of cmSLA implants at 33.7 (39.5) Ncm in group A (Table 2). At 3 and 6 weeks after implant insertion, the RTV of TSV MP-1 HA implants was significantly higher than that of cmSLA implants (*p* = 0.02 and *p* = 0.03, respectively). To account for the different ITV baselines, they were subtracted from RTV measurements to establish significant differences over time between implant systems. The change in RTV after accounting for the ITV baseline increased significantly for each implant over both healing times (*p* < 0.001; Table 2). However, this change was not significantly different between the two implant systems at three (*p* = 0.97) and six weeks healing time (*p* = 0.45), indicating progressive osseointegration and peri-implant stability of both implant systems.

### 2.4. Histological Evaluation

**Figure 2:**
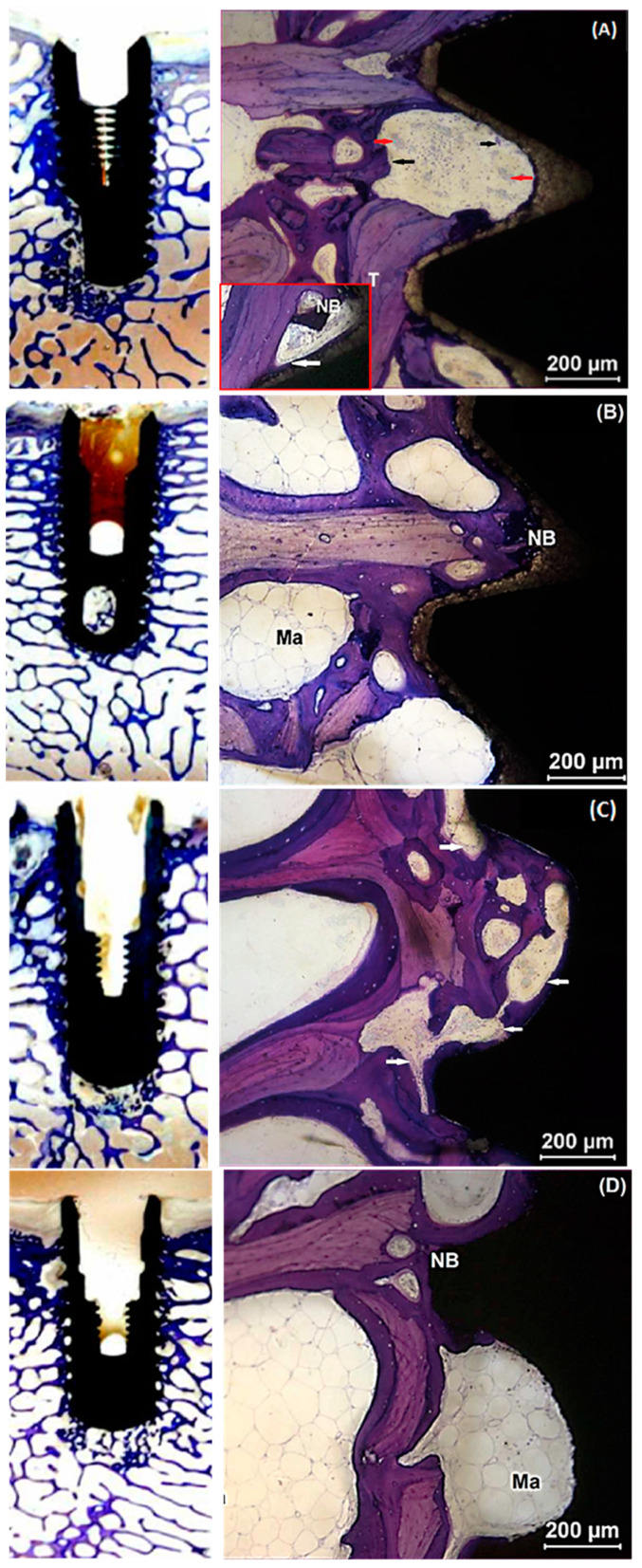
Representative histological images of TSV MP-1 HA (**A**,**B**) and cmSLA (**C**,**D**) implant surfaces at week 3 (**A**,**C**) and 6 (**B**,**D**). At 3 weeks, new mineralized bone (NB) is shown in direct apposition to the implant surface. The oval shapes are osteoclasts (black arrows) and the irregular shapes are blood vessels (red arrows). The inset in (**A**) and white arrows in (**A**,**C**) delineate the small cuboidal cluster of cells reminiscent of osteoblasts. At 6 weeks, the bone marrow (Ma) is more notable from the outline of the trabecular bone (T).

No adverse tissue responses were found with the TSV MP-1 HA implants at 3 and 6 weeks. At 3 weeks, native trabecular bone and a thin layer of unmineralized osteogenic tissue were observed directly in contact with the HA surface of the TSV MP-1 HA implant. The presence of osteoclasts and osteoblasts in the vicinity of newly formed bone indicated active bone remodeling. Osteoclasts were distinguished by their giant multinucleated cells and ruffled borders (Figure 2A). In contrast, osteoblasts were detected as smaller cuboidal-shaped cells that line up when secreting the bone matrix. The detection of osteoid, or unmineralized collagen tissue that stained light pink, was also an indication of the presence of a seam of osteoblasts nearby (Figure 2). Interstitial tissue at the bone-implant interface showed a continuous line of osteoblasts (Figure 2A inset image) and some osteoclastic activity (Figure 2A) indicative of active bone formation and remodeling. At 6 weeks, the newly formed trabecular bone appeared thicker, and the interstitial tissue at the implant interface showed a greater amount of new bone with predominantly mature marrow as compared to 3 weeks (Figure 2B).

Similarly, there was no evidence of any adverse tissue response to the cmSLA implants at 3 and 6 weeks. At 3 weeks, new woven bone was directly apposed to the threaded surface of the implant with trabecular elements observed close to the implant surface in the original bone bed. Appositional lamellar and new bone extended to trabecular elements. A continuous layer of osteoblasts and seam of osteoid on the newly formed bone at the bone-implant interface (intra-thread) was also detected (Figure 2C). At 6 weeks, a greater amount of new bone was observed at the bone-implant interface with a mature bone marrow, as compared to 3 weeks (Figure 2D).

### 2.5. Histomorphometry

The increased BIC% from 3 to 6 weeks healing time was not statistically significant for either TSV MP-1 HA (*p* = 0.15) or cmSLA implants (*p* = 1; Table 3). In group A, BIC% for TSV MP-1 HA at a median (IQR) of 92 (10) % and cmSLA of 87 (27) % were not significantly different (*p* = 0.15). However, the BIC% for TSV MP-1 HA, which had a median (IQR) of 98 (5) % was significantly larger as compared to cmSLA at 79 (17) % in group B (*p* = 0.01; Table 3). The BAF% of TSV MP1-HA was not significantly different from that of cmSLA across the 3- and 6-week healing times at the intra-thread (*p* = 0.20 and *p* = 0.42), adjacent 1 mm bone (*p* = 0.15 and *p* = 0.08), and the combined regions (*p* = 0.42 and *p* = 0.75), respectively (Table 3).

## 3. Discussion

This study was designed to determine peri-implant bone formation during early bone healing of a high crystalline HA-coated implant in the ovine femoral condyle model. The validity of the results was supported by comparison to a hydrophilic implant previously shown to result in early osseointegration in the tibiae of ovine [22] and oral cavity of canines [13,17,20]. In the ovine tibiae model, the efficacy of the cmSLA surface on the progress of early osseointegration was evaluated in comparison to the standard rough SLA surface at the 3- and 6-week healing time points. Histological analysis and implant stability tests exhibited superior bone contact and resistance to reverse torque forces in the cmSLA group, indicating that the hydrophilic surface of an implant resulting from chemical modification can improve the progression toward early bone healing [22]. However, the authors noted that the dense cortical bone structure of the tibia resulted in excellent outcomes for both cmSLA and SLA implants that may have explained their similarities in reverse torque measurements [22].

The ovine femoral condyle model in the present study was chosen for multiple reasons. Bone turnover and remodeling rates of the ovine are closer to that of humans when compared to other animal models [25]. In addition, bone outside the oral cavity would be less susceptible to variable influences caused by infection [23,24]. The medial side of the femoral condyle was chosen due to its higher weight-bearing capacity for implantation purposes as compared to the lateral side [27,31]. The femoral condyles of sheep mostly contain trabecular bone [27], which is a soft porous bone that is also prevalent in different areas of the oral anatomy [32]. Type IV trabecular bone is expected to show lower BIC% over comparable time points to that of cortical bone [33]. As a result, the ovine femoral condyles served as a challenging bone site to reveal robust and statistically relevant outcomes over those that may have been obscured due to either variability or small-scale influences under normal conditions.

Surface chemistry and topography of implants are key contributing factors in early peri-implant bone healing. For instance, roughened implant surfaces have been shown to display better activation of the molecular mechanisms involved in the initial stages of bone formation, such as blood-clot interaction, inflammation, angiogenesis, and cell migration [2]. Indeed, the rougher surface afforded by sandblasted and acid-etched implants resulted in a larger mean BIC% compared to anodized implants after 12 weeks implantation in the femoral bone of six rabbits in vivo [34]. Early osseointegration with the HA coating on titanium is supported through the following in vitro studies. Specifically, the HA coating on the TSV MP-1 HA implant has been shown to enhance the osteoconduction process and proposed to improve implant anchorage in bone by rendering the formation of biologically active hydroxyapatite [35]. Increased osteoblast adhesion has also been observed with increasing calcium amount present in the outer oxide layer of HA-coated titanium [36], and at the molecular level, it has resulted in enhanced attachment and expression of key osteogenic regulatory genes [37]. Despite the reported advantages of rough surface implants, the release of titanium particles is known to occur across implant types, including those with machined, rough, and coated surfaces [38]. However, the risk associated with the release of titanium particles from implants remains controversial for various reasons. In addition to the minimal evidence indicating an impact on the host, there are influences imposed by bone debris, surgery, and peri-implantitis treatment. The article by Marenzi et al. [39] concluded that the higher peaks and valleys produced through chemical etching could be more vulnerable to titanium wear during implant placement. Although the focus of our study related to outcome measures of early osseointegration, no adverse effects were noted during necropsy when examining the tissue around the implants in either the 3- or 6-week groups. It is evident from the SEM images of the cmSLA implants that the pits resulting from the acid-etching process were much smaller, at less than 10 µm, and superimposed within the larger craters from the blasting process. The SEM morphology of cmSLA implants indicated larger crater sizes ranging from 25–30 µm in depth and diameter as compared to the previous study in which the maximum valley depth was measured at much lower ranges for the media-blasted implants [39]. Thus, it is less likely that the additional acid-etching process would supersede the craters in height. Taken together, the cmSLA implants are not expected to result in any differences in particle release compared to other implant types.

The TSV MP-1 HA implants also differ from the cmSLA implants in macrogeometry resulting in a high insertion torque for the former due to the dimensions of the osteotomy based on the site preparation. The site preparation largely depends on the implant design, which determines the extent of lateral bone compression required for an increase in initial mechanical retention [40]. The ITV for each implant system represented adequate implant insertion according to the site preparation protocol provided by the manufacturer. Furthermore, ISQ measurements of both implant systems were similar and represented good primary stability with respect to the Osstell Scale. Primary stability reflects minimal micro-mobility of an implant anchored in bone when measured through RFA. The Osstell Scale is a range of empirically established ISQ values that depict levels of primary stability using a non-linear correlation to implant micro-mobility. The highest primary stability has been determined to be an ISQ of greater than or equal to 70 on the Osstell Scale. The discrepancy in baseline ITV measurements results from the greater influence that site preparation has on torque forces measured throughout the implant length. In contrast, ISQ resonance is predominantly localized to the coronal part of the implant and is thus, less likely to incur influences from different site preparations. To eliminate site preparation bias, baseline ITV measurements were subtracted from RTV outcomes for statistical comparisons within and between implant systems. The removal torque-to-failure testing, which measures shear forces at the bone-implant interface, was able to statistically differentiate the progression of peri-implant bone healing from baseline ITV over both healing times within each implant system. However, the changes in RTV over time were not significantly different between the two implants. The RFA, which measures the lateral stability of the implants, significantly increased with respect to the baseline ISQ over both healing times for cmSLA implants. A significant increase in ISQ occurred only at the 6-week healing time for the TSV MP-1 HA implants. Nevertheless, these changes in ISQ were not significantly different between the implant groups.

Removal torque forces are used as a biomechanical measure of bone anchorage to the implant. The greater reverse torque forces required to remove the implants are interpreted as an increase in the strength of osseointegration. However, using removal torque testing alone may not be adequate to assess the progression of osseointegration, as the underlying biomechanical phenomena in torque testing are very complex and can be influenced by multiple factors other than surface characteristics, such as implant macrogeometry, drilling protocols, and host response following implant insertion [41]. The discrepancy between RTV and ISQ was addressed by histological analysis as a direct measure of early bone healing. The histological evidence in the present study shows bone surrounding both TSV MP-1 HA and cmSLA implant surfaces during early peri-implant bone healing (Figure 2). However, histomorphometric measures of BIC% were significantly larger for TSV MP-1 HA compared to cmSLA implants at the 6-week healing time (Table 3). The comparison of BIC% at 6 weeks between the implant systems indicated greater contact osteogenesis around TSV MP-1 HA implants.

The significant increases from baseline with respect to ISQ and RTV measurements were not significantly different between the implant types as expected. However, BIC% was significantly larger for TSV MP-1 HA implants at 6 weeks compared to cmSLA implants. This indicated that BIC% was not particularly influential in ISQ and RTV measurements of mechanical stability. There were some limitations with the present study. Implant macrogeometry is known to play a stronger role in achieving optimum anchorage of the implant at initial insertion. In contrast, surface properties are more critical in achieving enhanced secondary stability through osseointegration during healing. However, the different macrogeometry of the two implant systems did not permit a direct comparison between absolute measures. Instead, changes over time were assessed to determine the trajectory of early peri-implant bone healing in both implant systems. Although the implants received a static or dynamic weight-bearing load from the skeletal structure of the animal, none were exposed to environmental influences, as is the case in the clinical situation with the oral cavity. Hence, different observations on the early healing behavior of the implant systems may result in the clinic. Though clinical studies are needed to further verify the clinical relevance of the findings reported herein, the outcomes of this study point to the possibility of early osseointegration of the TSV MP-1 HA implant.

## 4. Materials and Methods

### 4.1. Animal Model

The NIH guidelines for the care and use of laboratory animals (NIH Publication #85-23 Rev. 1985) were observed, and the Animal Welfare Committee (Flinders University, Australia) approved the study protocol. A total of 10 male sheep (Merino Ovis Aries) at 3 years of age and weighing approximately 57–71 kg were chosen for the surgeries. The animals were divided into 2 healing time groups of 3 weeks (Group A) and 6 weeks (Group B) with 5 sheep per group. Three implants were randomly placed in both medial femoral condyles for a total of 60 implants across healing time groups.

### 4.2. Implant Systems

**Figure 3:**
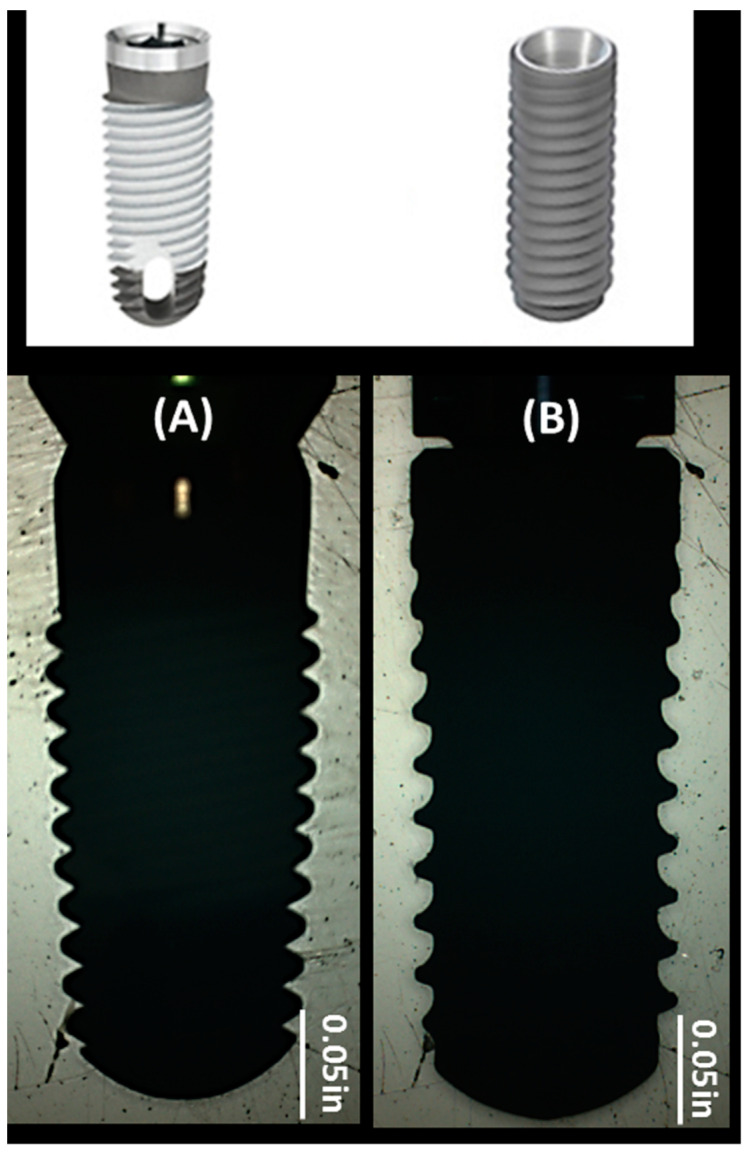
Macrogeometry of (**A**) TSV MP-1 HA and (**B**) cmSLA implant systems. The implants have different thread shapes and pitches. TSV MP-1 HA also has a vent in the apical region, which is not seen in the picture taken by the MicroVu system.

The 2 implant systems tested were of the same size (4.1 mm in diameter and 10 mm in length) but differed in macrogeometry (Figure 3). The Tapered Screw-Vent (TSV) MP-1 HA implant consisted of a triple-lead thread design that was a 60° V-shape with a thread depth of 0.4 mm. According to Sanz-Martín et al. [42], the standard tissue level Straumann implant consisted of a reverse buttress thread with a 1.2 mm pitch, 0.3 mm depth, and an upper and lower angle of 16° and 36°, respectively. The same was assumed for the Straumann bone-level cmSLA implants, as the main difference in macrogeometry with respect to the tissue-level implant was the transmucosal collar. The TSV MP-1 HA-coated implant was made of extra-low interstitial (ELI) titanium (Ti) alloy. The implant had a microtextured surface created by grit-blasting with HA powder and was washed in nitric acid (HNO_3_) and distilled water without any acid etching process. The HA coating was then formed on the cleaned surface via a plasma spraying method followed by a hydrothermal treatment step that renders the TSV MP-1 HA implant with a highly crystallized HA coating [4]. The cmSLA implant was made of grade 4 commercially pure (CP) Ti, and the surface was sandblasted with large-grit aluminum oxide (Al_2_O_3_). This was followed by acid etching with hydrochloric acid (HCl) and sulfuric acid (H_2_SO_4_), rinsing under a nitrogen environment, and immersing in a saline solution, which imparts its hydrophilic surface [17]. Surface morphology and elemental analysis of the implants were conducted using a scanning electron microscope (SEM; EVO-60, Zeiss, Württemberg, Germany) equipped with an energy dispersed X-ray (EDX) spectrometer (Bruker AXS Quantax 4010, Mikroanalysis GmBH, Berlin, Germany). Furthermore, the average surface roughness values (R_a_) were quantitatively measured using an optical profilometer with a 50X objective, 1.0X field of view, and x and y resolutions of 60.83 µm and 31.87 µm, respectively (NT1100 Optical Profiling System, Veeco, Tucson, AZ, USA). A total of 10 measurements were equally divided between the superior and inferior thread flanks and tips of each implant system.

### 4.3. Surgical Procedure and Euthanasia

**Figure 4:**
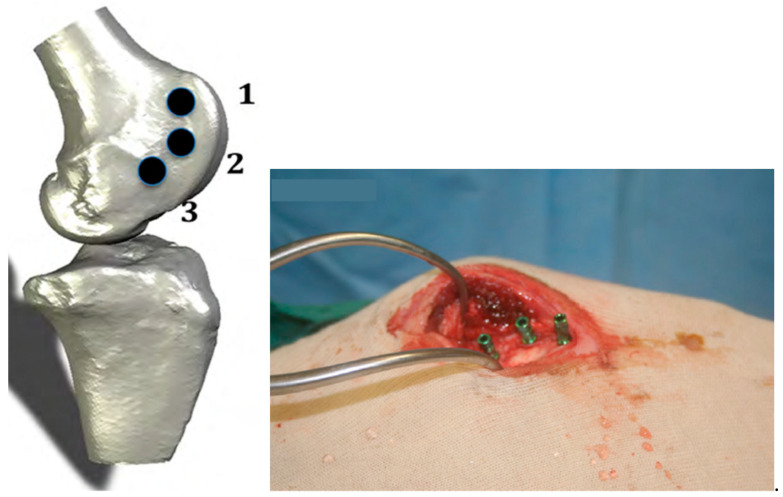
(**Left**) Three implants randomly inserted into the femoral condyle of the sheep in the positions 1, 2, and 3. (**Right**) The green components are the transfer mounts that are left in place for photo capturing purposes and were removed and replaced by cover screws prior to closing the wound.

The surgical protocol has been previously described in detail elsewhere [43]. Briefly, surgery commenced with perioperative analgesia using a combination of Xylazine (0.05 mg/kg), Thiopentone (20 mg/kg), and Isoflurane (2% in oxygen) administered systemically via a jugular catheter. The antibiotic, Cephalosporin (Kefzol, 30 mg/kg), was also administered intravenously before the surgery and 2 days postoperatively. Three implants were randomly placed in both medial femoral condyles at an even spacing of 1.0 ± 0.5 cm (Figure 4). The incision was held open using self-retaining retractors that reached down onto the condyle. Osteotomies were prepared with a series of incremental sizes of internally irrigated drills under copious amounts of saline to reduce heat generation at the surgical site. During implant placement, the ITV was measured using a calibrated digital torque device (Sturtevant Richmont, Carol Stream, IL, USA). The implants were placed at the crestal level according to the instructions for use provided by each manufacturer. A cover screw was placed to limit tissue growth inside the implant, and primary wound closure was achieved with a 3-0 chronic gut suture. At 3 and 6 weeks of healing, 5 sheep per time point were euthanized with an intravenous barbiturate overdose of Pentobarbital Sodium (9.75 g in a 30 mL bolus).

### 4.4. Resonance Frequency Analysis

Early peri-implant bone stability was assessed via the ISQ using resonance frequency analysis [44]. The ISQ measurements were obtained at 8 equidistant positions around the implant via a calibrated Osstell device (Osstell AB, Gothenburg, Sweden) that works together with an RFA coupling component attached to the implants. The cover screws were removed from the implants, and different coupling component sizes were used for TSV MP-1 HA implants (Smart Peg #32, Osstell AB) and cmSLA (Smart Peg#54, Osstell AB). Measurements were performed at implant placement (baseline) and following euthanasia at 3 and 6 weeks of healing time.

### 4.5. Torque Measurement

Removal torque-to-failure of 36 randomly selected implants were measured at 3 and 6 weeks healing time (*n* = 9 per implant system and time point) following RFA and euthanasia. A calibrated torque driver connected to a digital torque gauge (BGI, Mark-10, Copiague, NY, USA) was positioned in the internal connection of the implant and turned counterclockwise to measure the maximum removal torque value (RTV). The results were stored in a data acquisition system for analysis (MESUR^™^ gauge, version 1.5, Mark-10, Copiague, NY, USA).

### 4.6. Histology and Histomorphometry

**Figure 5:**
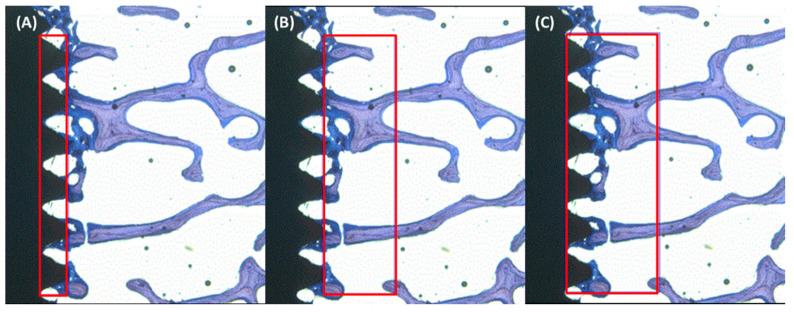
Histomorphometric measurements were performed by defining the ROI to calculate BAF% in (**A**) intra-threads, (**B**) adjacent 1 mm host bone bed, and (**C**) the total of intra-threads plus adjacent 1 mm host bone bed regions.

The remaining 24 undisturbed implants were explanted using a trephine drill at 3 and 6 weeks healing time (*n* = 6 implant system and time point). The retrieved implant specimens were immersed in 10% formalin buffered solution for 48 h to fixate, followed by dehydration in alcohol and embedding in polymethylmethacrylate for sectioning. A cutting system (Makro Trennsystem; Exakt Apparatebau AG, Norderstedt, Germany) was used to prepare 3 undecalcified sections parallel to the longitudinal axis of the resin-embedded specimen. One side of the section was attached to a flexible polycarbonate plastic slide, while the other was ground to approximately 50 µm of target thickness using a bench-top grinder (Buehler, Lake Bluff, IL, USA). Toluidine blue and basic fuchsin were used to stain the sections, which were examined using a light microscope (Olympus BH-2, Olympus Optical Company, Tokyo, Japan). The photomicrographs of each section were taken at 18 kV for 10 s using a cabinet X-ray system (Faxitron Corp., Tucson, AZ, USA). The bone-to-implant contact (BIC) was calculated using Scion Image Analysis software (Scion Corp, Frederick, MD, USA) by tracing the entire length of the implant surface area in direct contact with mineralized bone tissue. The measured value was expressed as a percentage of the axial implant surface. The BAF% was measured across the entire implant length with respect to 3 regions of interest (ROI) encompassing the implant intra-threads, adjacent 1 mm host bone bed, and both regions combined. The amount of bone within the ROI was measured by calculating the percentage of area inside the ROI occupied by bone (Figure 5) [45].

### 4.7. Statistical Analysis

Descriptive statistics and statistical analysis were obtained through a computer-based software program (Minitab 16 Minitab Inc., State College, PA, USA). The Shapiro–Wilks test was used to assess whether the data significantly differed from a normal distribution. As some groups of measurement across time points or implant types did not pass the normality test, a nonparametric Kruskal–Wallis test was used to determine significant differences at *p* ≤ 0.05. All measurements were reported as the median and interquartile range accordingly.

## 5. Conclusions

The study showed enhanced early osseointegration of up to 6 weeks using a plasma-sprayed HA-coated implant of high crystallinity in which favorable histological evidence resulted in an ovine medial femoral condyle model. In summary, fundamental differences in primary implant stability measured by ISQ were not detected between the two implant systems. In addition, changes over time in ISQ or RTV were not significantly different between the two implant systems. However, significantly higher BIC% was observed at 6 weeks for the TSV MP-1 HA implant as compared to the cmSLA implant. Therefore, TSV MP-1 HA may be a potential candidate for accelerated early bone healing. A similar comparison was made in a canine mandible study, whereby no significant differences were observed in bone volume and BIC% between cmSLA and another implant with discrete crystalline deposition (DCD) of calcium phosphate particles [46]. Thus, either the surface energy or coating morphology can impact early peri-implant bone healing. The bone mineral-like hydroxyapatite coating of metal implants adds to the repertoire of dental treatment by promoting osseointegration in sites with lower density bone [30].

## Figures and Tables

**Table 1:** Surface elemental composition determined by EDX and average surface roughness values (R_a_) measured by optical profilometry.

Implant Type	Element Analysis (Atomic %)	Surface Roughness (R_a_) (µm)
**TSV MP-1 HA**	C (1.67), O (37.34), P (18.84), Ca (42.15)	4.63 ± 0.83
**cmSLA**	O (2.57), F (0.44), Na (14.74), Cl (21.73), Ti (60.52)	1.57 ± 0.24

**Table 2:** (**A**) Median (IQR) ISQ and (**B**) median (IQR) torque values for TSV MP-1 HA and cmSLA systems measured at 0 (insertion), 3 weeks (Group A), and 6 weeks (Group B) post-implantation (*n* = 9 per implant system and time point). Darker rows were used to determine within-group and between-group differences from baseline. The *p* values in bold show statistically significant differences.

**(A) ISQ Median (IQR)**			
	**TSV MP-1 HA**	**cmSLA**	**Between Groups**
**Group A (Week 3)**			
Week 0	69.2 (2.8)	70.0 (6.9)	*p* = 0.24
Week 3	71.5 (9.1)	72.5 (5.1)	***p* = 0.01**
Difference (Week 3 − 0)	0.1 (8.2)	2.2 (2.8)	*p* = 0.08
Week 3 (Within Group)	*p* = 0.65	***p* = 0.02**	--
**Group B (Week 6)**			
Week 0	66.5 (5.6)	68.4 (8.7)	*p* = 0.37
Week 6	70.8 (4.4)	72.4 (7.2)	*p* = 0.15
Difference (Week 6 − 0)	6.2 (8.8)	2.6 (8.7)	*p* = 0.75
Week 6 (Within Group)	***p* = 0.03**	***p* = 0.05**	--
**(B) Torque Median (IQR)**			
	**TSV MP-1 HA**	**cmSLA**	**Between Groups**
**Group A (Week 3)**			
Week 0 (ITV)	102.7 (61.2)	33.7 (39.5)	***p* = 0.01**
Week 3 (RTV)	158.5 (36.6)	120.5 (82.4)	***p* = 0.02**
Difference (Week 3 − 0)	74.6 (66.8)	63.5 (56.3)	*p* = 0.97
Week 3 (Within Group)	***p* < 0.001**	***p* < 0.001**	--
**Group B (Week 6)**			
Week 0 (ITV)	86.7 (45.8)	35.4 (19.8)	***p* = 0.04**
Week 6 (RTV)	267.9 (44.6)	212.2 (62.0)	***p* = 0.03**
Difference (Week 6 − 0)	181.6 (42.5)	165.1 (99.0)	*p* = 0.45
Week 6 (Within Group)	***p* < 0.001**	***p* < 0.001**	--

**Table 3:** Histomorphometric data comparison for TSV MP-1 HA and cmSLA systems at 3 and 6 weeks of healing period (*n* = 6 per implant system and time point). The *p* values in bold show a statistically significant difference.

**Table 3. BIC% & BAF%**	**Week 3 Median (IQR)**	**Week 6 Median (IQR)**	**Within Groups**
**BIC%**			
TSV MP-1 HA	92 (10)	98 (5)	*p* = 0.15
cmSLA	87 (27)	79 (17)	*p* = 1
Between Groups	*p* = 0.15	***p* = 0.01**	--
**BAF% at intra-threads**			
TSV MP-1 HA	51 (16)	50 (12)	*p* = 0.87
cmSLA	47 (11)	47 (14)	*p* = 0.75
Between Groups	*p* = 0.20	*p* = 0.42	--
**BAF% at adjacent 1 mm host bone**			
TSV MP-1 HA	44 (14)	43.9 (21)	*p* = 1
cmSLA	38 (14)	42.0 (17)	*p* = 0.63
Between Groups	*p* = 0.15	*p* = 0.08	--
**BAF% total of intra-threads and adjacent 1 mm host bone**			
TSV MP-1 HA	46 (13)	45 (21)	*p* = 1
cmSLA	41 (8)	43 (19)	*p* = 1
Between Groups	*p* = 0.42	*p* = 0.75	--

## Data Availability

The data presented in this study are not publicly available but can be requested from the corresponding author.
